# Impact of the coronavirus disease 2019 on the diagnoses of Hansen's disease in Brazil

**DOI:** 10.1590/0037-8682-0251-2021

**Published:** 2021-07-23

**Authors:** Nelson Pereira Marques, Nádia Carolina Teixeira Marques, Iberto Medeiros Cardozo, Daniella Reis Barbosa Martelli, Edson Gomes de Lucena, Eduardo Araújo Oliveira, Hercílio Martelli

**Affiliations:** 1 Universidade Estadual de Campinas, Faculdade de Odontologia de Piracicaba, Piracicaba, SP, Brasil.; 2 Universidade José do Rosário Vellano, Centro de Reabilitação de Anomalias Craniofaciais, Alfenas, MG, Brasil.; 3 Universidade José do Rosário Vellano, Departamento de Odontopediatria, Alfenas, Minas Gerais, Brasil.; 4 Faculdade FUNORTE, Escola de Medicina, Instituto de Ciências da Saúde, Montes Claros, MG, Brasil.; 5 Universidade Estadual de Montes Claros, Programa de Pós-graduação em Ciências da Saúde, Montes Claros, MG, Brasil.; 6 Universidade Federal da Paraíba, Departamento de Clínica e Odontologia Social, João Pessoa, PB, Brasil.; 7 Universidade Federal de Minas Gerais, Faculdade de Medicina, Belo Horizonte, MG, Brasil.; 8 University of California, San Diego, La Jolla, CA, USA.

**Keywords:** COVID-19, Hansen's disease, Pandemics

## Abstract

**INTRODUCTION:**

Coronavirus disease 2019 (COVID-19) has limited the access of patients with Hansen's disease (HD) to care due to changes in routine health services.

**METHODS:**

To ascertain this, we compared the number of HD cases diagnosed before and after the COVID-19 pandemic.

**RESULTS:**

The decrease in HD cases in Brazil reached 18,223 (-48.4%), corresponding to an average reduction of 1,518 cases per month during the COVID-19 pandemic.

**CONCLUSION:**

Therefore, effective measures should be implemented to minimize the damage and the consequent negative health impact of COVID-19 on the care of HD patients.

The World Health Organization (WHO) declared coronavirus disease 2019 (COVID-19), which is caused by severe acute respiratory syndrome coronavirus 2 (SARS-CoV-2), a pandemic in March 2020^1^. In Brazil, the first COVID-19 case was reported on February 26, 2020. Just a year after that, 18.7 million people have been diagnosed with COVID-19 and approximately 524,000 deaths have been reported^2^. The high infection and hospitalization rates caused by SARS-CoV-2 have led to several disturbances in health systems around the world, including the Brazilian Unified Health System (SUS)^3^ ^,^ ^4^.

In response to the increase in the number of COVID-19 cases, non-urgent consultations and hospital admissions have been discouraged or postponed, leading to a change in the priorities of hospital care^4^ ^,^ ^5^. The high infectivity of SARS-CoV-2 raises important questions about COVID-19 risk groups^6^. Mortality seems to be higher in men, older adults, people who do not receive assistance from health services, and people with chronic diseases, such as hypertension, diabetes, coronary heart disease, and pulmonary obstructive diseases^6^ ^,^ ^7^. However, there is still a lack of information regarding how COVID-19 affects people with chronic infectious diseases^6^. 

The pandemic has limited the access of patients with Hansen's disease (HD) to care^5^. HD is an infectious, chronic, and granulomatous disease caused by *Mycobacterium leprae* (with high infectivity and low pathogenicity), which was quite prevalent in Europe during the 16th century and currently affects 200,000 people globally^1^ ^,^ ^8^. Clinically, HD cases are classified as paucibacillary (PB), with up to five lesions on the body, or multibacillary (MB), with more than five lesions; the latter has greater potential for transmission due to the high bacillary load and the spread of bacillus through the upper airways^1^ ^,^ ^8^. 

HD mainly affects the peripheral nervous system, skin, and mucosa, with symptoms varying depending on the immune response of the patient to bacterial infection. During transmission by droplets, *Mycobacterium leprae* affects the nasal mucosa capillary endothelial cells and, subsequently, reaches the Schwann cells of the nervous system. This way, infection of the olfactory bulb can occur and lead to olfactory dysfunction, which is clinically characterized as hyposmia or anosmia and is similar to some of the symptoms of COVID-19^8^.

HD is curable; however, it can progress to severe neural damage and lead to amputation of the limbs if not treated early; this makes early diagnosis even more important for preventing worse outcomes. Besides, Mahato et al. (2020) affirmed that the prevalence of HD is higher among people of low social status; these are the same people most affected by the measures for mitigating the COVID-19, such as restrictions of non-essential services and recommendations of social isolation, which favor the increase in social inequalities. Because of this condition, several people cannot maintain appropriate hygienic measures to prevent SARS-CoV-2 infection, including frequent washing of hands and the use of alcohol gel and masks, among other measures. If contaminated by Hansen's bacillus, they may develop severe leprosy reactions and possibly co-infection with SARS-CoV-2^5^ ^,^ ^7^.

To further ascertain this, we evaluated the number of cases of HD diagnosed between January 2010 and December 2020 using data collected from the National Disease Notification System (SINAN)^9^. We also compared the data for the pre- and peri-pandemic periods. The diagnoses of the disease were already declining within 2010-2019, as shown by the Mann-Kendall trend test (-0.6444, P = 0.012). Figure 1 shows a decline in HD diagnoses between 2010 and 2015 with a tendency to stabilize during the subsequent years, followed by a steep drop in 2020. Considering the average number of newly diagnosed cases of HD in 2010-2019 compared with 2020, the reduction was consistent across all five Brazilian macroregions, ranging from 41% in the Midwest to 56.4% in the Southeast macroregion. The decrease in the number of Brazilian cases reached 18,223 (-48.4%), corresponding to an average reduction of 1,518 cases per month during the COVID-19 pandemic (Table 1). Table 2 shows the comparison between the mean rates during the pre-pandemic years and 2020 across Brazilian geographic macroregions and the entire country. The rates of HD diagnoses significantly decreased during the pandemic period throughout Brazil (IRR= 0.51, 95% CI 0.50-0.52, P<0.0001).

**FIGURE 1:**
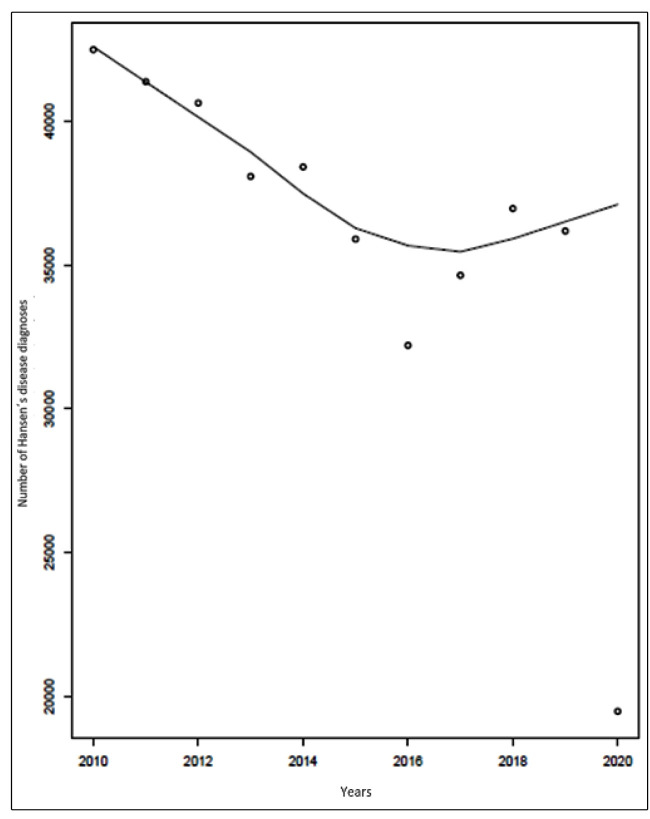
Plot of the number of diagnoses of Hansen’s disease in Brazil by the Brazilian public health system (2010-2020).

**TABLE 1:** Comparison of the diagnoses of Hansen's disease in Brazil by the Brazilian public health system within 2010-2019 and in 2020.

Macroregions of Brazil	2010-2019	2020	Difference (%)
North	7,409	3,876	-3,533 (-47.7)
Northeast	15,877	8,190	-7,687 (-48.4)
Southwest	5,657	2,408	-3,249 (-57.4)
South	1,349	638	-711 (-52.7)
Midwest	7,409	4,366	-3,043 (-41.0)
**Total**	**37,701**	**19,478**	**-18,223 (-48.4)**

**Source:** Notifiable Diseases Information System - Sinan Net (http://portalsinan.saude.gov.br/).

**TABLE 2:** Incident cases of Hansen's disease per million population in Brazilian macroregions within 2017-2019 and in 2020.

Macroregions of	2010-2019	2020		
Brazil	Incidence rate	Incidence rate	Incidence ratio	p*
	(95%CI)	(95%CI)	(95%CI)	
North	413	216	0.52	< 0.001
	(403 - 422)	(209 - 223)	(0.50 - 0.54)	
Northeast	277	143	0.51	< 0.001
	(273 - 281)	(140 - 146)	(0.50 - 0.53)	
Southwest	65	27	0.42	< 0.001
	(63 - 67)	(26 - 28)	(0.40 - 0.44)	
South	45	21	0.47	< 0.001
	(43 - 48)	(19 - 23)	(0.43 - 0.52)	
Midwest	46	27	0.59	< 0.001
	(44 - 47)	(26 - 28)	(0.56 - 0.61)	
**Total**	**181**	**94**	**0.51**	**< 0.001**
	**(179 - 183)**	**(92 - 95)**	**(0.50 - 0.52)**	

HD: Hansen's disease; CI: Confidence interval; * *p-*value: obtained by chi-square Chi^2^-statistic.

In conclusion, our study showed a reduction in the number of HD cases diagnosed in Brazil during the pandemic. Concerns about the diagnoses and treatment of neglected tropical diseases have heightened during the pandemic period, possibly due to the reduction of financial support and human resources^10^. Therefore, effective measures, including the advancement of COVID-19 vaccination, dissemination of information about protective measures by health care professionals, and awareness of the population about the importance of HD control should be urgently prioritized to minimize the negative impact of COVID-19 on health services for HD.

## References

[1] Schmitz V, dos Santos JB (2021). COVID-19, leprosy, and neutrophils. PLoS Negl Trop Dis.

[2] Ministério da Saúde (MS) (2021). Painel de casos de doença pelo coronavírus 2019 (COVID-19) no
Brasil.

[3] Remuzzi A, Remuzzi G (2020). COVID-19 and Italy: what next?. Lancet.

[4] Simões e Silva AC, Oliveira EA, Martelli-Junior H (2020). Coronavirus Disease Pandemic Is a Real Challenge for
Brazil. Front Public Health.

[5] Mahato S, Bhattarai S, Singh R (2020). Inequities towards leprosy-affected people: A challenge during
COVID-19 pandemic. PLoS Negl Trop Dis.

[6] Santos VS, Quintans-Júnior LJ, Barboza WS, Martins-Filho PR (2021). Clinical characteristics and outcomes in patients with COVID-19
and leprosy. Eur Acad Dermatol Venereol.

[7] Antunes DE, Goulart IMB, Goulart LR (2020). Will cases of leprosy reaction increase with COVID-19
infection?. PLoS Negl Trop Dis.

[8] Abdelmaksoud A, Gupta SK (2020). Management of leprosy patients in the era of
COVID-19. Dermatol Ther.

[9] Ministério da Saúde (MS) (2021). Sistema de Informação de Agravos de Notificação - Sinan Net.

[10] de Souza DK, Picado A, Bieler S, Nogaro S, Ndung'u JM (2020). Diagnosis of neglected tropical diseases during and after the
COVID-19 pandemic. PLoS Negl Trop Dis.

